# Our Editorial Chit Chat

**Published:** 1869-04

**Authors:** 


### Our Editorial Chit Chat.

### The Velocipede.

A Dr. Van Wyck of New York, attempts to bring his name prominently before the public by writing articles for the press against the use of the velocipede.

He says, "the small, hard seat comes in contact with one of the most tender portions of the anatomy." Doubtless the Doctor has not forgotten his boyhood days when his mother applied her slipper to the "tender portion of his anatomy," for being so foolish *then* as he is striving to be now.

His prattle about "stranguary, inflammation of the testes and disease of the prostate," being caused by riding the velocipede, is all bosh.

We believe it to be one of the most delightful and healthful means of exercise ever presented to the public. Almost every muscle of the body is brought into play in propelling and balancing the machine. It expands the chest—invigorates the lungs—employs the mind—develops the muscles—and affords a pleasant and exhilarating means of exercise.

We advise every physician, lawyer, minister, merchant, clerk, or other parties who lead a sedentary life, to use a velocipede. Ride it before breakfast an hour, ride it to your place of business, and then ride it home again. It will invigorate and strengthen you—give you an appetite for your meals, and you will at once declare that you feel better for it. Try it, as we have done, and don't rely on the opinion of some old fogy, who has neither the courage nor disposition to tackle the machine.

### Danger from Postage Stamps.

When you have a postage stamp enclosed in a letter to prepay answer, *never moisten it with your tongue or lips.* We have recently treated a gentleman for *chancre* of the lip, who contracted the disease by licking postage stamps that were enclosed to him by his correspondents.

There can be no doubt about the matter, as the disease was fairly traced to one of the gentleman's correspondents who has long been a sufferer from syphilis. Therefore, we charge our readers never to apply an enclosed postage stamp to their mouths—you do not know through whose hands it has passed, and it may be the means of inoculating you with some foul disease. Use a wet sponge or cloth with which to moisten the stamp, and all danger will be avoided.

### Duty of Post Masters.

We occasionally have a package of BISTOURIES returned to us, by some blundering Post Master, marked "refused", without any reason being assigned for such refusal, or any mark upon the package by which we can possibly judge from whence it came. So that our only alternative is to look through *twenty thousand names* for the ones returned us by an ignorant Post Master. It is the duty of a Post Master, when a journal is refused by a subscriber, to write his name upon a printed slip, provided them by the Department for that purpose, stating the *reason* for such refusal, and having the name of his office plainly written or stamped upon said slip. We hope that if any more of our journals should be returned to us, that Post Masters will take the trouble to write the name of the Post Office upon them, so that we may be spared the trouble of searching an entire day for the names.

### Poisoning from Hair Dye.

Most of the "hair invigorators" sold in the shops "for imparting a smoothness and gloss to the hair, and for restoring it to its natural color," &c., are composed of preparations of lead and sulphur.

We have seen several cases of almost fatal poisoning recorded in our medical journals, since our last issue, the result of the free use of these patent hair preparations. The lead is absorbed through the scalp and carried into the general circulation, giving rise to all the symptoms of lead poisoning. It will be wise for those who are addicted to the use of these nostrums to be more careful in their use, and to see that they are not too freely applied, else serious consequences might ensue.

**A New Demand.**

The general use of the velocipede this summer, will make a new demand upon our Merchants. When their lady customers approach their establishments mounted upon a velocipede, it will be necessary not only for the clerks to open the door for the fair ones to enter, but also to throw out a plank in order that they can reach the store room in safety with their steeds. An extra room should also be provided, where the vehicles can be stored while their riders are making purchases. Let us see who will be the first to supply this demand.

**Enlarge the Treasury.**

Will not Mr. Beecher enlarge the treasury box at the Opera House? As it is now, we are compelled to carry a piece of shingle with us to the Sunday Meetings, with which to thrust our shinplasters into the box. This is a great inconvenience to us, and wife complains that the shingle does more injury to our pocket than the loss of the money. Hope that those having the matter in charge will enlarge the box.

**The Sphygmograph.**

This instrument, but recently perfected, is now rapidly coming into favor with our best physicians. It is a machine for measuring the pulse, and it does it with so much accuracy and has revealed to the physician so many new facts relative to a diseased circulation, that its universal adoption by every scientific physician, is sure to follow.

The instrument consists of a metallic frame, about an inch and one half wide, by six inches long, upon which is constructed a small "clock work" for moving the disk upon the long arm of a peculiarly constructed lever which traces the pulse wave. The machine is strapped to the wrist, the short arm of the lever rests upon the radial artery, the clock work is set in motion and while the rising and falling of the pulse imparts its motion to the short arm of the lever, the long arm—which terminates in a pen—writes the movement plainly upon the disk. With each beat of the heart the lever is raised twice, which explains that two pulsations occur in the radial artery for every beat of the heart. The second pulsation is much weaker than the first, but is always present in a healthy pulse. In fever, the pulse undergoes a great change—sometimes the second beat is entirely wanting, while in other forms of disease several pulsations are given to one beat of the heart. Those who have become proficient in the use of this little instrument are able to detect many diseases that before could not be so readily recognised. In short, it is one of the most useful discoveries that has yet been made for the use of the physician, and it will not be a great while before "feeling the pulse" will be among the things that have been.

**"Thinker and Observer."**

If the correspondent, by the above *nom de plume*, who has written us two long letters from Worcester, N. Y., will make his articles shorter, and write but upon one side of the page, we will publish them.

**The Simese Twins.**

Appealing from the opinion of the Edinburg doctors that it would be very dangerous to cut them asunder, are on their way to consult the medical fraternity of Paris.—*Exchange*,

And Professor Nilaton of Paris says they can be safely sundered. The operation should have been performed long ago, for we believe Prof. Nilaton to be right. It is unfortunate that they did not consult a yankee surgeon years ago, for had they done so, they would now be occupying separate sleeping apartments with their wives.

**New Operating Room.**

We have leased the rooms next our Institution for a term of years, and Dr. ELDRIDGE, (who, by the way, is an excellent landlord,) has fitted them up in magnificent style for us. We now have the best arranged operating rooms in the State—everything being as neat and convenient as money can make it. We have also had a new entrance made to the Institute—our patients will now find us one stairway further up street than formerly.

**The Reason Why.**

Some of or old subscribers may fail to get their BISTOURIES this quarter, for the reason that we have cut down our lists at all Post Offices where we have over twenty subscribers.

Only those who send fifty cents for the BISTOURY, or join a club for it, will receive it *regularly* in future. If we have any left after supplying our *paying* subscribers, we will distribute them as before—otherwise not. Bear this in mind, and send in your name and money at once.

